# Development and Characterization of Novel Combinations and Compositions of Nanostructured Lipid Carrier Formulations Loaded with Trans-Resveratrol for Pulmonary Drug Delivery

**DOI:** 10.3390/pharmaceutics16121589

**Published:** 2024-12-12

**Authors:** Iftikhar Khan, Sunita Sunita, Nozad R. Hussein, Huner K. Omer, Abdelbary Elhissi, Chahinez Houacine, Wasiq Khan, Sakib Yousaf, Hassaan A. Rathore

**Affiliations:** 1 School of Pharmacy and Biomolecular Sciences, Liverpool John Moores University, Liverpool L3 3AF, UK; gradsunita@outlook.com (S.S.); s.s.yousaf@ljmu.ac.uk (S.Y.); 2 College of Pharmacy, Hawler Medical University, Erbil 44001, Iraq; nozad.hussein@hmu.edu.krd (N.R.H.); huner.omer@hmu.edu.krd (H.K.O.); 3 Department of Pharmaceutical Sciences, College of Pharmacy, QU Health Sector, Qatar University, Doha 2713, Qatar; aelhissi@qu.edu.qa; 4 School of Pharmacy and Biomedical Sciences, University of Central Lancashire, Preston PR1 2HE, UK; chahinez.houacine1@gmail.com; 5 Faculty of Engineering and Technology, Liverpool John Moores University, Liverpool L3 3AF, UK; w.khan@ljmu.ac.uk

**Keywords:** nanostructured lipid carriers, cancer, anti-cancer, pulmonary system, aerosolization

## Abstract

Background/Objectives: This study aimed to fabricate, optimize, and characterize nanostructured lipid carriers (NLCs) loaded with trans-resveratrol (TRES) as an anti-cancer drug for pulmonary drug delivery using medical nebulizers. Methods: Novel TRES-NLC formulations (F1–F24) were prepared via hot, high-pressure homogenization. One solid lipid (Dynasan 116) was combined with four liquid lipids (Capryol 90, Lauroglycol 90, Miglyol 810, and Tributyrin) in three different ratios (10:90, 50:50, and 90:10 *w*/*w*), with a surfactant (Tween 80) in two different concentrations (0.5 and 1.5%), and a co-surfactant, soya phosphatidylcholine (SPC S-75; 50 mg). Results: Amongst the analyzed 24 TR-NLC formulations, F8, F14, and F22 were selected based on their physicochemical stability when freshly prepared and following storage (4 weeks 25 °C), as well as in terms of particle size (<145 nm), polydispersity index (PDI; <0.21) and entrapment efficiency (>96%). Furthermore, F14 showed greater stability at 4 and 25 °C for six months and exhibited enhanced aerosolization performance, demonstrating the greater deposition of TRES in the later stages of the next-generation impactor (NGI) when using an air-jet nebulizer than when using an ultrasonic nebulizer. The F14 formulation exhibited greater stability and release in acetate buffer (pH 5.4), with a cumulative release of 95%. Conclusions: Overall, formulation F14 in combination with an air-jet nebulizer was identified as a superior combination, demonstrating higher emitted dose (ED; 80%), fine particle dose (FPD; 1150 µg), fine particle fraction (FPF; 24%), and respirable fraction (RF; 94%). These findings are promising in the optimization and development of NLC formulations, highlighting their versatility and targeting the pulmonary system via nebulization.

## 1. Introduction

Advancements in inhalational drug delivery systems have resulted in successful applications in the prophylaxis and treatment of localized lung diseases (e.g., asthma, chronic obstructive pulmonary disease, cystic fibrosis, pulmonary hypertension, and cancer) [1]. However, due to the added advantage of bypassing first-pass metabolism, this route has started to attract growing interest for the treatment/management of systemic diseases [2,3]. The lung is distinguished by its large contact surface area (~100 m^2^), thin epithelium (i.e., 0.1–0.2 µm), and rich blood supply. Drug bioavailability following inhalation can further be promoted by low enzymatic degradation, high membrane permeability, and rapid onset of action [1,4,5]. For these reasons, the development of advanced delivery systems, particularly for those that use nanotechnology, has seen a surge in interest to exploit this non-invasive route of administration [6,7]. Specifically, nanoparticles are known to offer a large surface to volume ratio, high drug encapsulation, higher biodistribution, controlled and site-specific drug release [8,9], potentially low systemic toxicity [10,11], and better formulation stability during nebulization [12].

Lipid nanoparticle drug delivery systems provide considerable benefits, including easy preparation, high biodegradability, scale-up feasibility, reduced toxicity, and targeted delivery [12,13]. Various types of lipid-based formulations have achieved the successful delivery of both hydrophilic and hydrophobic compounds [14]. There are several types of lipid nanoparticles including liposomes [14,15]. Prominent examples of liposome products include Doxil^®^, which is a doxorubicin-loaded PEGylated liposome formulation designed for the treatment of breast and ovarian cancer [14,16]. Other lipid nanoparticle formulations include niosomes [17,18], transfersomes [1], ethosomes, and surfactosmes [19].

Advancements in technology have led to the development of second-generation lipid nanoparticles, which started with solid lipid nanocarriers (SLNs), followed by third-generation nanocarriers, referred to as nanostructured lipid carriers (NLCs). SLNs have been vastly explored for delivering various types of agents (e.g., iron oxide, tachnitium-99, and insulin) [20,21]. SLNs are biocompatible delivery systems having low toxicity and a smaller size (50–1000 nm) and can be easily manufactured on a large scale [22]. Despite these advantages, SLNs have shortcomings such as poor drug loading capacity (attributed to the crystalline nature of the solid lipid), drug leakage (following polymorphic transition), lipid particle growth, as well as particle aggregation and solidification. Consequently, NLCs were introduced to improve drug loading, stability, and compatibility. NLCs are combinations of solid and liquid lipids and hence possess a less crystalline matrix and imperfect solidified core, which prevent the leakage of the drug [23].

TRES, a natural polyphenol (chemically known as 3,4′,5-trans-trihydroxystilbene), is a biologically active compound and possesses multiple biological activities (i.e., anti-inflammatory, anti-oxidant, anti-tumor, as well as neuro- and cardioprotective effects) [11,24,25,26]. Despite the promising biological effects, it has low systematic availability due to rapid clearance, limiting its clinical application [27]. Moreover, TRES is hydrophobic molecule with slow dissolution and low oral bioavailability [28]. NLC formulations can improve absorption and protect against the rapid metabolism of drugs. The success of pulmonary therapy is not only dependent on the pharmacology of the inhaled drug but also on the site and extent of drug deposition in the respiratory tract [29]. Thus, for pulmonary targeting using lipid-based formulations, these must possess optimum aerodynamic properties such as aerosol median aerodynamic diameter and polydispersity, which determine the fine particle dose (FPD), and fine particle fraction (FPF) of the inhaled aerosol. Nebulizers are highly recommended for the delivery of liquid formulations (i.e., solutions or suspension) into the pulmonary system. The in vitro assessment of the aerodynamic diameter of aerosol particles released from nebulizers can be analyzed via a next-generation impactor (NGI) [30].

The present study aimed to formulate, optimize, and characterize TRES-NLC formulations for their deposition in an NGI. TRES-NLC formulations were studied based on their novel combination and composition ratios of different types of solid lipid, liquid lipid, surfactant, and co-surfactant ingredients. These were assessed in terms of particle size, PDI, zeta potential, drug entrapment efficiency, and percentage recovery. The formulations were stored at 4 and 25 °C for six months to analyze their stability at different temperatures. Based on their physicochemical properties and the stability studies, the optimum TRES-NLC formulation was selected for an aerosolization study using air-jet and ultrasonic nebulizers. Additionally, in vitro release was studied to determine the effect of two media with different pH values at 37 °C on the release profile of TRES.

## 2. Materials and Methods

### 2.1. Materials

HPLC-grade acetonitrile, formic acid, absolute ethanol, tetrahydrofuran and ammonium molybdate were purchased from Fischer Scientific Ltd., Loughborough, UK. Trans-resveratrol (C_14_H_12_O_3_) was procured from Manchester Organics (Runcorn, UK). Tripalmitin (Dynasan 116; C_51_H_98_O_6_) and caprylic triglyceride (Miglyol 810; C_21_H_40_O_5_) were gifted by IOI Oleochemical GmbH (Witten, Germany). Propylene glycol monocaprylate type II (Capryol 90; C_11_H_22_O_3_) and propylene glycol monolaurate (Lauroglycol 90; C_15_H_30_O_3_) were provided by Gattefose (Ascot, Berkshire, UK). Tributyrin (C_15_H_26_O_6_) was bought from Across Organics (New Jersey, NJ, USA). Tween 80 (C_64_H_124_O_26_) was purchased from Sigma Aldrich, Gillingham, UK. Soya phosphatidylcholine S-75 grade (SPC; C_42_H_80_NO_26_P) was gifted by Lipoid GmbH, Ludwigshafen, Germany.

### 2.2. Preparation and Optimization of Nanostructured Lipid Carriers (NLCs)

Combinations of one solid lipid and four different types of liquid lipids were used at three different ratios (10:90, 50:50, and 90:10 *w*/*w*) to form the lipid phase. Each combination of lipid phase was then formulated with two different percentages of the surfactant Tween 80 (i.e., 0.5 and 1.5%) to prepare 24 different TRES-NLC formulations via hot, high-pressure homogenization. Dynasan 116 was used as the solid lipid, whereas the four liquid lipids employed included Capryol 90, Lauroglycol 90, Miglyol 810, and Tributyrin. Tween 80 and SPC S-75 were employed as the surfactant and co-surfactant in all formulations. Each TRES-NLC formulation was prepared using three different phases, i.e., liquid phase, aqueous phase, and drug phase (Table 1).

The lipid phase (4% of total formulation) was prepared by heating a solid lipid (Dynasan 116) and one of the liquid lipids (Capryol 90, Lauroglycol 90, Miglyol 810, or Tributyrin) in a ratio of 10:90 (200 mg:1800 mg) and melted at 70 °C (~10 °C above the phase transition temperature of the solid lipid) using digital hotplate magnetic stirrer (ADS-HP-NT, Asynt, IKA laboratories equipment, Germany). Similarly, other combinations of solid lipid and liquid lipids i.e., 50:50 *w*/*w* (1000 mg:1000 mg) and 90:10 *w*/*w* (1800 mg:200 mg), were used to prepare separate lipid phases. The aqueous phase was prepared separately by preheating deionized water at 70 °C (sufficient to prepare 50 mL of formulation), using Tween 80 at two different concentrations (0.5% (250 mg) and 1.5% (750 mg)), which were combined using a digital hotplate magnetic stirrer. The drug phase was prepared by dissolving 500 mg of TRES and 500 mg of SPC-S75 in 10 mL of ethanol. Subsequently, 1 mL (containing 50 mg of TRES and 50 mg of SPC-S75) of this phase was transferred into the preheated molten lipid phase. This was followed by the addition of the preheated aqueous phase into the lipid phase using continuous stirring (1250 rpm) for 15 min in order to prepare a dispersion system (i.e., microemulsion). For uniform homogenization, the obtained dispersion was subjected to homogenization (T18 Ultra-Turarax; IKA, Königswinter, Germany.) for 3 min at 10,000 rpm. The resultant uniform microemulsion was homogenized to a nano size by probe-sonication (Qsonica Probe Sonicator, Newtown, CT, USA) for 5 min using an amplitude intensity of 60%. TRES-NLC formulations were successfully obtained following cooling of the solid lipid to room temperature (25 °C).

### 2.3. Particle Size and Zeta Potential Analysis

For the calculation of size and zeta potential, 0.5 mL of TRES-NLC formulation was diluted with 10 mL of water. Particle size and polydispersity index (PDI) of TRES-NLC formulations were measured by dynamic light scattering (DLS) using a Zetasizer (Malvern Zetasizer Nano series, Malvern, Worcestershire, UK) at 25 °C. Moreover, after aerosolization, TRES-NLC formulations deposited in various stages of the NGI were washed with water and subjected to vortex-mixing for 1 min (Labnet International, Edison, NJ, USA), followed by particle size analysis. A Zetasizer was also employed to determine the zeta potential of the TRES-NLC particles using laser doppler velocimetry (LDV).

### 2.4. Determination of Entrapment Efficiency of Drug

The entrapment efficiency of the TRES-NLC formulations were determined via Millipore filters (3 kDa; Fisher Scientific, Loughborough, UK). To determine the unentrapped or free drug (TRES), 0.5 mL of TRES-NLC formulation was pipetted into a Millipore centrifuge filter that was fitted into an Eppendorf tube. Bench centrifugation (Spectrafuge 24D, Labnet International, Edison, NJ, USA) was then conducted at 5900 rcf for 15 min. The clear filtrate at the bottom of the Eppendorf tube was collected (centrifuge filters allowed the free drug to pass through their pores, while retaining the TRES entrapped in the NLCs) and concentration of free TRES was determined using high-performance liquid chromatography (HPLC) (Agilent 1200 Series Instrument, Waldborn, Germany). To quantify the total drug, 1 mL of TRES-NLC formulation was diluted with tetrahydrofuran, followed by running through HPLC. An HPLC instrument was used at 25 °C at a wavelength of 239 nm. A reverse-phase Luna C-18 column (Phenomenex, Torrance, CA, USA) with a 5 µm particle size, 4.6 mm internal diameter, and 250 mm length was used as the stationary phase. The flow rate was set t 1 mL/min with an injection volume of 20 µL. HPLC analysis was performed using a mobile phase (comprised of 0.1% formic acid and acetonitrile).

The entrapment efficiency of TRES was calculated using Equation (1), and percentage recovery was calculated using Equation (2).

(1) $$Entrapment\;efficiency\;(\%)=\frac{Totaldrug-Unentrappeddrug}{Total\;drug}\times 100$$

(2) $$Recovery\left(\%\right)=\frac{Practical\;amount\;of\;drug\;obtained\;from\;HPLC\;calibration\;curve}{Theoretical\;amount\;(amount\;of\;drug\;added\;during\;preparation)}\times 100$$

### 2.5. TRES-NLCs Formulation Stability Study

The initial stability studies of the TRES-NLC formulations were conducted for particle size, PDI, zeta potential, and entrapment efficiency in order to compare the physicochemical properties between the freshly prepared formulations as well as following storage at 25 °C for four weeks. All formulations were kept in an amber-colored glass bottles (25 mL), and the temperature was maintained throughout this study. Particle size analysis and zeta potential measurements were conducted as described in Section 2.3 and entrapment efficiency as described in Section 2.4.

Based on the stability study of the F1–F24 formulations, only selected TRES-NLC formulations were used for subsequent studies. These selected formulations were then characterized (particle size, PDI, zeta potential, entrapment efficiency, and drug recovery) over a period of six months under two different temperatures (4 and 25 °C).

### 2.6. Morphology Study via Transmission Electron Microscopy (TEM)

One drop of the TRES-NLC formulation and two drops of a negative stain ammonium molybdate were mixed and transferred onto a carbon-coated copper grid (400 mesh) (TAAB Laboratories Equipment Ltd., Aldermaston, UK) and then allowed to dry for 1 h. Samples were then placed in the TEM instrument (Morgagni 268D, EFI, MegaView, Brno, Czech Republic), and the morphology of the TRES-NLC formulations was observed.

### 2.7. Aerosolization Study of TRES-NLC Formulation Using Next-Generation Impactor (NGI)

The NGI (Copley Scientific, Nottingham, UK) was operated with a critical airflow controller (Copley TPK 2000, Copley Scientific, UK) and vacuum pump (Copley flow HCP5, Copley Scientific, UK). The airflow was adjusted to 15 L/min (European Pharmacopeia general chapter 2.9.44) for the aerodynamic assessment of nebulized aerosols.

Before aerosolization, the NGI and its collecting compartments were all kept in a fridge for 90 min at 5 °C. Two nebulizers were used: an air-jet (PARI GmbH Tourboboy 5 air-jet, Starnberg, Germany) and an ultrasonic nebulizer (Rechargeable Ultrasonic Inhaler MY-520B, China). All empty collection trays, induction port (mouthpiece), and nebulizers were weighed separately prior to starting nebulization. TRES-NLC formulations (5 mL) were pipetted into the nebulizer and weighed. The nebulizer was positioned in front of the induction port before starting nebulization. After completed nebulization (i.e., when the nebulizer stopped producing aerosols), the nebulization time (continuous formation of aerosols) and sputtering time (the intermittent formation of aerosols until no further aerosol formation) were recorded. The mass output (Equation (3)) and mass output rate (Equation (4)) were calculated. Moreover, the quantity of formulation deposited into each stage was determined via HPLC analysis.

(3) $$Mass\;output\left(\%\right)=\frac{Weight\;of\;nebulized\;formulation}{Weight\;of\;formulation\;present\;in\;nebulizer\;before\;nebulization}\times 100$$

(4) $$Mass\;output\;rate\left(\frac{mg}{min}\right)=\frac{Weight\;of\;nebulized\;formulation}{Complete\;nebulization\;time}$$

The emitted dose (ED) was the total amount TRES in the formulation emitted from the nebulizer. The fine particle dose (FPD) was the mass of particles less than 5 µm in diameter of the total ED. The fine particle fraction (FPF) was the fraction of particles (i.e., less than 5 µm in diameter) correlated to the emitted mass, and the respirable fraction (RF) represented the fraction of particles entering stages 2–7. ED (Equation (5)), RF (Equation (6)), FPD (Equation (7)) and FPF (Equation (8)) were calculated using the following equations:

(5) $$ED\left(\%\right)=\frac{Initial\;mass\;in\;nebulizer-Final\;mass\;remaining\;in\;nebulizer}{Initial\;mass\;in\;nebulizer}\times 100$$

(6) $$RF\left(\%\right)=\frac{Fine\;particle\;dose}{Total\;particle\;mass\;on\;all\;stages}\times 100$$

(7) FPD=Mass of drug deposited on stage 2 through 7

(8) $$FPF(\%)=\frac{Fine\;particle\;dose}{Initial\;particle\;mass\;loaded\;in\;nebulizer}\times 100$$

### 2.8. Study of TRES Released from NLC Formulations

The release profile of TRES from the NLCs (1 mg/1 mL) was studied using dialysis tubing (a cut-off MWCO; 3500 Daltons) placed in the dissolution apparatus II USP (Varion Instrument, Cary, NC, USA). The dialysis bag was selected based on its pore size, which should ideally be 5–10 times larger than the molecular weight of TRES (i.e., 228 Daltons) [7]. This allowed the TRES molecules only to pass through the dialysis bag, while retaining the NLC particles due to their large size. TRES-NLC formulations (5 mL) were sealed in the dialysis bag and placed in release media (900 mL), and the rotation speed of the paddles was adjusted to 100 rpm at 37 °C. The time-dependent release of the drug was investigated in two different media: acetate buffer (pH 5) and deionized water (pH 7). Samples (1 mL) were withdrawn from each media at specific time intervals (0.5, 1, 2, 3, 4, 5, and 24 h) and replaced each time with the same volume of drug-free media. TRES alone (5 mg/5 mL) was also employed as a control for comparison. The amounts of TRES released were estimated using HPLC (Section 2.4).

### 2.9. Statistical Analysis

Analysis of variance (ANOVA) and Student’s *t*-tests were performed using SPSS software (IBM SPSS Statistics 28.0) in order to determine whether the difference was significant between the groups. A *p* value lower than 0.05 indicated the difference was statistically significant. All experiments were conducted in triplicate using three different batches.

## 3. Results and Discussion

### 3.1. Initial Investigation and Selection of TRES-NLC Formulations

The initial selection of TRES-NLC formulations was conducted based on particle size, PDI, zeta potential, and entrapment efficiency. Comparisons were made between freshly prepared formulations and formulations stored at 25 °C for four weeks.

In the TRES-NLC formulations prepared using Capryol 90 as a liquid lipid, larger particles were observed, attributed to particle aggregation; thus, a significant difference (*p* < 0.05) was noted in particle size between the freshly prepared TRES-NLCs and the TRES-NLCs stored at 25 °C for four weeks. This may have been related to the higher viscosity of Capryol 90, which may have prevented oil droplet emulsification, resulting in larger nanoparticles [31]. Lauroglycol 90 is a medium-chain mono-glyceride, and Miglyol 810 and Tributyrin are medium- to short-chain triglycerides; when medium- to shorter-chain glycerides were employed in the formulations, enhanced stability in terms of particle size was observed, linked to the lower viscosity of the employed glycerides (Table 2) [32]. Moreover, upon comparing the particle size and PDI of the freshly prepared TRES-NLC formulations, it was observed that the formulations containing higher liquid lipid (i.e., 10:90, solid to liquid lipid *w*/*w* ratio) and surfactant concentrations (i.e., 1.5%) had smaller particle size and PDI when compared to the formulations with low concentrations of liquid lipid and surfactant (produced large particles and a broader PDI) (Table 2). F22 showed greater potential based on its stability, particularly in terms of particle size and growth of the NLCs. In contrast, F20 exhibited a higher tendency for aggregation and sedimentation during storage, which could lead to stability issues upon long-term storage.

Differences in particle size could be attributed to the solid lipid concentration, as this may affect the melting/solidification process during NLC production (Table 2), forming agglomerates when higher concentrations are used. Despite probe sonication, the agglomerates resisted breakdown, resulting in a wide PDI [13]. The effect of solid lipid concentration is evidenced in research conducted by Das, et al. [33] and Gokce, et al. [34], where increases in particle size have been observed. This increase in solid lipid concentration in the NLC formulations was posed to increase the dispersion viscosity, resulting in size increases [35,36]. A concentration-dependent relationship between surfactant and particle size has also been observed, with higher concentrations being associated with smaller particles [37]. This trend is also supported by previous findings [36,38].

Based on the particle size and PDI, TRES-NLC formulations F8, F14, and F22 (containing liquid lipid Lauroglycol 90, Miglyol 810, and Tributyrin) were selected, where the particle size of the freshly prepared samples was less than 150 nm, and the PDI value was less than 0.20. Particle sizes of 150 nm were used as a guide to compare formulation stability during storage conditions. Moreover, the formulations with a smaller particle size and lower PDI were preferred due to their large surface area as well as dose uniformity. Moreover, particle size is the major determinant in the deposition and distribution of inhaled drugs within the pulmonary system to achieve localized effects. Smaller particles possess the ability to reach the small airways and alveolar epithelium compared with larger particles with a wider PDI. This is attributed to the ability of nanoparticles to be incorporated into the “respirable” nebulized droplets. No significant difference (*p* > 0.05) was observed among the three selected formulations in terms of particle size or PDI when a comparison was conducted between freshly prepared formulations and formulations stored for four weeks at 25 °C (Table 2). Unlike the particle size findings, the zeta potentials were similar (*p* > 0.05) between the fresh TRES-NLC formulations and samples stored at 25 °C for four weeks (Table 2). The zeta potential values were more than −20 mV, due to the presence of the free hydroxyl group of the liquid lipids in the formulations [37].

Furthermore, no significant difference (*p* > 0.05) was observed in the entrapment efficiency (Table 2). The high entrapment efficiency was associated with using higher amounts of liquid and solid lipid (2000 mg in total), offering a large area for the drug to be encapsulated. There are other studies that have employed a higher amount of TRES and still achieved higher entrapment efficiencies. A study conducted by Mathew et al. [1] also used TRES (50 mg) with much lower amounts of solid and liquid lipids (i.e., 252 mg in total) in NLC formulations but still achieved a 95% entrapment efficiency, which is thus unlikely attributable to saturation. In the same study, liposomes and transfersomes were prepared with the same amounts of lipid phase and TRES, which still achieved high entrapment efficiency, demonstrating the ability of TRES to lodge within the vesicles. Similar results were also found by Houacine et al. [37] with regard to the entrapment efficiency of TRES in NLC formulations. The higher entrapment efficiency of TRES may be related to its high miscibility with lipids (i.e., solid and liquid) and surfactant. In general, the incorporation of higher concentrations of lipids may also reduce particle crystallinity and cause disorder in the crystal lattice, which allow greater accommodation of the drug (offering high solubility in the lipid matrix), leading to higher drug loading capacity and hence higher entrapment efficiency (>95%) (Table 2). Additionally, higher concentrations of liquid lipid and solid lipid may account for imperfections within the crystal matrix, providing more space in which the drug molecules can lodge [12,39,40]. The effect of liquid lipid on drug entrapment (i.e., increased with increasing concentration) has been previously reported [12,41,42,43].

Overall, it was found that higher concentrations of liquid lipid and surfactant in TRES-NLC formulations may be responsible for the higher drug entrapment, smaller particle size, and lower PDI. Therefore, based on the characterization studies, formulations F8, F14, and F22 were selected for further studies.

### 3.2. Surface Morphology Study of TRES-NLC Formulations

**Figure 1:**
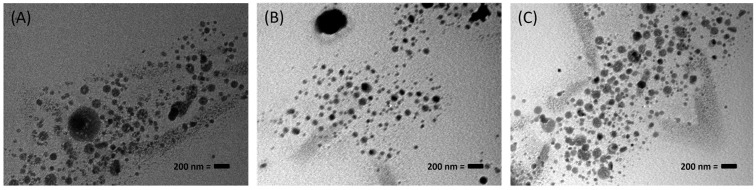
Transmission electron microscopy (TEM) images of TRES-NLC formulations (with 200 nm scale bar): (**A**) F8, (**B**) F14, and (**C**) F22. These images are typical of the three different batches.

When the selected TRES-NLC formulations (F8, F14, and F22) were investigated for particle morphology, they were found to be spherical, within the nanosize range (Figure 1).

### 3.3. Particle Size Analysis of TRES-NLC Formulations in NGI Stages

**Figure 2:**
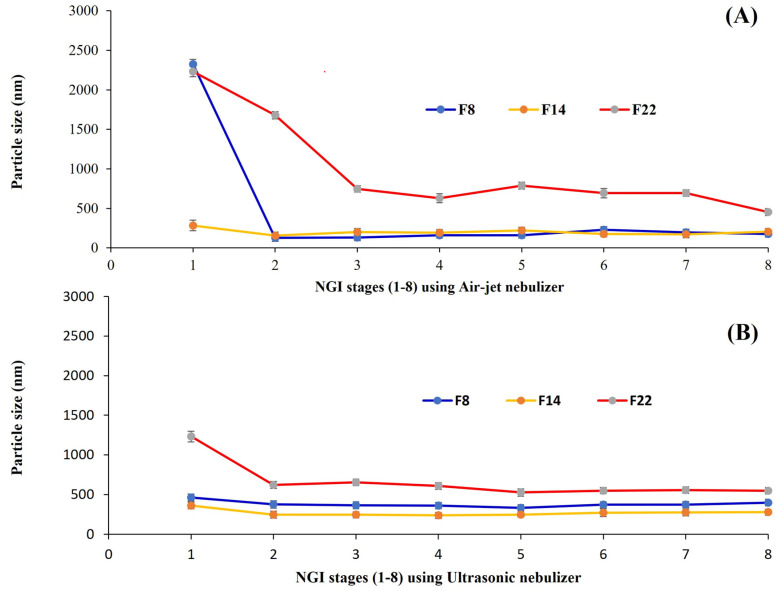
Deposition and analysis of aerosol droplets containing TRES-NLC particles of various formulations (F8, F14, and F22) in the various stages of a next-generation impactor (NGI) using (**A**) air-jet and (**B**) ultrasonic nebulizers at an airflow rate of 15 L/min. Data are presented as mean ± SD, n = 3.

Aerosolization studies were conducted on the selected TRES-NLC formulations (i.e., F8, F14, and F22). Using the air-jet and ultrasonic nebulizers, the aerosol deposition of TRES-NLC formulations in the NGI was analyzed in terms of particle size. After aerosolization, a decreasing trend in particle size was observed when moving from stage 1 to stage 8 in the NGI (Figure 2); thus, larger droplets/particles were deposited in the initial stages, and smaller particles were deposited in the advanced stages of the NGI [12,44]. It is important to consider the inertial action of aero-dispersed particles, i.e., particles accelerate at a comparatively high speed under the influence of compressed air (in air-jet nebulizers) when compared to the piezoelectric crystal vibration (employed in ultrasonic nebulizers). Therefore, it is suggested that air stream/aerosols containing the droplets/particles are accelerated through the nozzle of the nebulizers, causing comparatively stronger abrupt movement of the particles in air-jet nebulizers than in ultrasonic nebulizers. Consequently, particle separation occurs based on the differences in the inertia of the particles (which depends on their size and speed) [45]. Thus, stage 1 demonstrated deposition of larger particles, which was observed more prominently when the air-jet nebulizer was used (Figure 2A).

According to the analysis of the TRES-NLC formulation deposition in the NGI stages, the performance of formulation F14 demonstrated greater stability and consistency compared to formulations F8 and F22 (Figure 2). Moreover, the particle size of formulation F14 was significantly smaller (*p* < 0.05) than that of the F8 and F22 formulations (in all eight stages of the NGI when the ultrasonic nebulizer was used) (Figure 2B).

### 3.4. TRES-NLC Formulation (F8, F14, and F22) Stability Study

TRES-NLC formulations F8, F14, and F22 were studied for stability via storage for six months at 4 or 25 °C. Particle size, PDI, Zeta potential, drug entrapment efficiency and drug recovery were determined to evaluate formulation stability at months 2, 4 and 6. It was noted that F8, F14, and F22 had significantly larger (*p* < 0.05) particle sizes at each time point when compared to their freshly prepared formulations. This instability related to particle size was significantly more pronounced (*p* < 0.05) at 25 °C than at 4 °C, which was also confirmed by the higher PDI values (Table 3). Higher temperatures caused migration of the surfactant molecules (Tween 80, SPC S-75) from the oil/water interface, causing particle coalescence [46]. Lower temperatures (e.g., 4 °C) are more favorable for the storage of nanoemuslions containing polysorbate surfactants (e.g., Tween 80). Additionally, it has been reported that the oxidative degradation of TRES is inhibited at 4 °C [47]. Similar nanoparticle stability at 4 °C has been reported by previous researchers [42].

Zeta potential analysis and drug entrapment studies showed no significant difference (*p* > 0.05) between the freshly prepared and stored formulations (regardless of temperature) (Table 3). However, it is important to consider that slight changes in zeta potential values and entrapment efficiency may be related to storage temperature, where the temperature may marginally affect the crystallinity of lipid nanoparticles, which may cause minor disturbances in the electrical double diffuse layer of the formulation [48]. Notably, drug recovery was significantly affected at 4 and 6 months, especially at 25 °C, when compared to the freshly prepared TRES-NLC formulations (Table 3). TRES is not stable at alkaline pH, high temperatures, and during light exposure since the drug is prone to rapid degradation. Thus, drug recovery was significantly lower than for the freshly prepared TRES-NLC formulations. Additionally, TRES degradation was also found by Trela and Waterhouse [49], who reported the stability of TRES in buffers having pH 1, 3.5 or 7 upon storage for 28 days. Moreover, Liazid, et al. [50] reported that TRES can be degraded when exposed to high temperatures. The results from the current study were also confirmed and found to be in agreement with those of a previous study [47], where formulations were more stable at 4 °C than at 25 °C.

Overall, F14 demonstrated higher stability in terms of particle size, PDI, zeta potential, drug entrapment efficiency, and drug recovery (Table 3), as well as formulation deposition in various stages of the NGI (Section 3.3). TRES-NLC formulation F14 is comparatively more stable as it is composed of Miglyol 810 (liquid lipid), which is a medium-chain triglyceride, facilitating emulsification and reduced formulation aggregation; even when used at higher concentrations, the formulation was stable. These findings were also confirmed by Marzec, et al. [51], where lipid nanoparticles containing Miglyol 810 as a liquid lipid demonstrated greater stability. These findings indicate that the combination of Dynasan 116 and Miglyol 810 is compatible, producing stable NLC formulations for the delivery of TRES. Therefore, F14 was selected for subsequent studies.

### 3.5. Nebulization Performance of TRES-NLC Formulations

#### 3.5.1. TRES-NLC Formulation F14 Deposition in Various Stages of NGI

**Figure 3:**
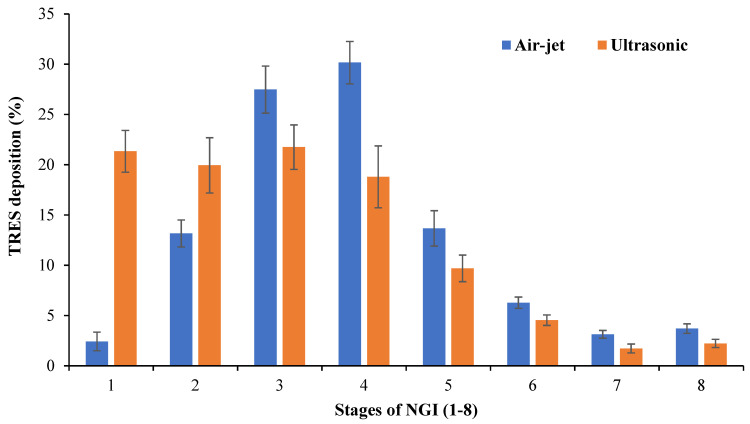
Deposition of TRES using TRES-NLC formulation F14 in various stages (1–8) of NGI via air-jet and ultrasonic nebulizers. Data are mean ± SD, n = 3.

According to the post-aerosolization analysis of the TRES-NLC formulation F14, the air-jet nebulizer demonstrated a lower deposition of TRES in the initial stages of the NGI and higher deposition in the middle to lower stages, which was significantly higher (*p* < 0.05) than that of the ultrasonic nebulizer (Figure 3). This could be attributed to the high velocity of the air compressed by the air-jet nebulizer, depositing larger droplets (containing a small mass of the formulation) in the initial stage and propelling smaller droplets (higher mass) in the later stages of the NGI [12]. Additionally, the presence of the baffle within the air-jet nebulizer plays an important role in TRES-NLC deposition. The smaller droplets generated by the air-jet nebulizer may escape the baffle, whereas larger droplets hit the baffle with high velocity, converting them into smaller droplets for inhalation, accounting for their deposition in the middle to later stages of the NGI, suggesting higher suitability for deep lung deposition. Similar results were observed when using the air-jet nebulizer, where two airflow rates (i.e., 15 and 60 L/min) were employed for beclometasone deposition [12]. The ultrasonic nebulizer produced significantly higher (*p* < 0.05) deposition of TRES in the early stages and lower deposition in the later stages of the NGI (Figure 3). This trend (opposite to the deposition trend of the air-jet nebulizer) may be related to the inconsistent and larger droplets formed by the ultrasonic nebulizer, resulting in deposition in the early stages.

#### 3.5.2. Nebulization Time of F14

**Figure 4:**
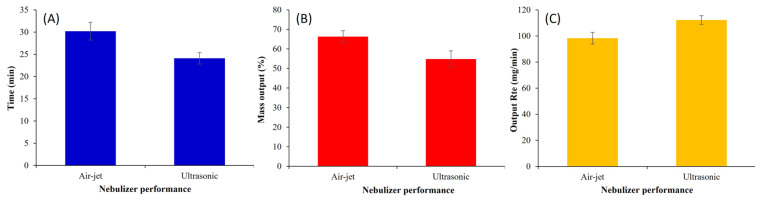
Nebulization performance, including the (**A**) nebulization time to “dryness” (min), (**B**) mass output (%), and (**C**) output rate (mg/min) of TRES-NLC formulation F14. Data are mean ± SD, n = 3.

The nebulization time to “dryness” using the TRES-NLC formulation F14 was significantly higher (*p* < 0.05) for the air-jet nebulizer compared to the ultrasonic device (Figure 4A), agreeing with earlier findings reported by Harvey, et al. [52].

The longer nebulization time for the air-jet nebulizer may be attributed to the latent heat of vaporization; this decreases the temperature of the nebulizer fluid, which in turn increases the fluid viscosity and decreases the nebulizer output. By contrast, the piezoelectric crystals of the ultrasonic nebulizer generate energy that increases the temperature of the nebulizer fluid, decreasing the content viscosity and shortening the nebulization time [7,53]. It is important to consider the design of the nebulizers, as air-jet nebulizers contain a baffle at the top of the venturi nozzle; as it produces fine droplets under the influence of highly compressed air flow, the smaller droplets escape for inhalation. By contrast, larger droplets impact the baffle and side walls, deflecting back into the nebulizer reservoir for further atomization into fine droplets; this increases the time needed to complete nebulization [54]. Overall, it can be concluded that the longer nebulization time of the air-jet nebulizer is associated with the deposition of higher drug amounts in the later stages of the NGI.

#### 3.5.3. Nebulization Mass Output and Mass Output Rate of TRES-NLC Formulation

It is important to note that 100% mass output is not achievable after the total nebulization time; thus, some residual content remains in the nebulizer reservoir at the end of nebulization. Mass output represents the quantity of aerosolized formulation estimated via the gravimetric technique (by weighing nebulizers before and after nebulization) [55]. The air-jet nebulizer demonstrated significantly higher (*p* < 0.05) mass output (66.28 ± 3.03%) of the TRES-NLC formulation F14 when compared to the ultrasonic nebulizer (54.85 ± 4.23%) (Figure 4B). This difference in mass output was attributed to the dead/residual volume. The ultrasonic nebulizer retained higher residual volume, as well as generated heat during nebulization [5,56], causing particle fusion/aggregation. Similar findings were presented by Elhissi, et al. [57], where air-jet nebulizers were observed to exhibit higher mass output (i.e., less residual mass).

The mass output rate is a function of nebulization time. A significantly higher (*p* < 0.05) output rate was exhibited by the ultrasonic nebulizer when compared to the air-jet nebulizer (Figure 4C). Similar findings of higher mass output and lower mass output rate by the air-jet nebulizer have been demonstrated in previous studies [12,58].

#### 3.5.4. Evaluation of ED, FPD, FPF, and RF of TRES-NLC Formulation F14

The total amount of TRES emitted and deposited in the various stages of NGI is referred to as the ED. A significantly higher (*p* < 0.05) ED was found when employing the air-jet nebulizer compared to the ultrasonic device (Table 4).

Upon analysis of the FPD and FPF, the air-jet nebulizer was found to be more efficient in delivering and depositing the TRES-NLC formulation F14 in the “deep” NGI stages compared to the ultrasonic nebulizer (Table 4). Moreover, similar results were noted for the RF upon comparing the nebulizers (Table 4). It is important to note that droplets with an aerodynamic size of less than 5 µm possess the ability to travel further into the later stages of the NGI; therefore, these droplets are considered to be within the “respirable” fraction of the nebulized formulation [54]. However, droplets in excess of this size have a higher chance of deposition in the initial stages of the NGI (i.e., induction port and stage 1), which is related to inertial impaction (larger droplet possess lower maneuverability compared to smaller droplets) when employing an airflow rate of 15 L/min [12]. The smaller droplet size generated from air-jet nebulizers is also attributed to the built-in baffles positioned above its “venture” nozzle. During nebulization, as droplets are generated, smaller droplets are able to evade the baffle system for inhalation, whereas larger droplets impact the baffles, deflecting the primary aerosol back into the reservoir for further fragmentation into secondary aerosol droplets of “respirable” size.

Thus, although the air-jet nebulizer was associated with a prolonged nebulization time, it managed to generate smaller droplets that traveled deeper to the later stages of the impactor. The impact of droplets on the baffle in an ultrasonic nebulizer is milder (due to the distance between the generated droplets and the baffle position) than in an air-jet nebulizer. Thus, the generated droplets are propelled without restriction to the NGI despite their broad size distribution. Thus, the ultrasonic nebulizer is associated with a shorter nebulization time, with aerosol deposition across all NGI stages. Overall, it was found that the air-jet nebulizer exhibited superior aerosolization performance and hence is more appropriate for aerosolizing TRES-NLC formulations.

### 3.6. In Vitro Release Study of TRES-NLC Formulation F14

**Figure 5:**
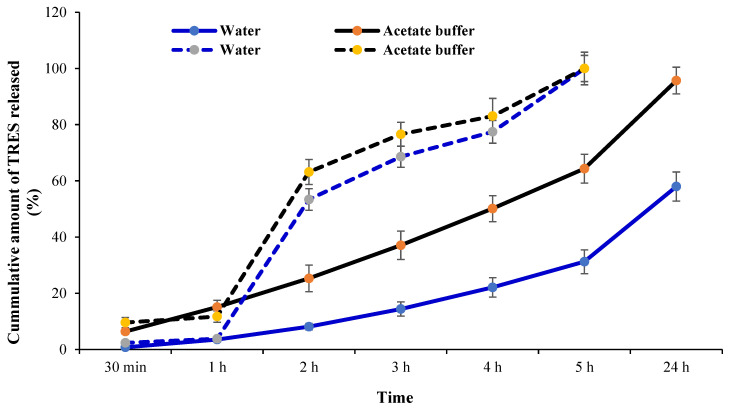
In vitro release of TRES from TRES-NLC formulation F14 (solid lines) and TRES as a free drug alone (doted lines) in two dissolution media, including water (pH 7) and acetate buffer (pH 5.4). Data are mean ± SD, n = 3.

The sustained release profile of formulation F14 was analyzed in two different dissolution media, which were water (pH 7) (i.e., the suggested medium for the formulation) and acetate buffer (pH 5.4) (the pH physiologically reflective of the lungs in the presence of cancerous growth). Over a period of 24 h, the maximum release of TRES from NLC formulation F14 was found to be 58.02% and 95.79% in water and acetate buffer, respectively (Figure 5). It is noteworthy that TRES possesses phenate ions in its structure, which are susceptible to electrophilic attack and forms phenoxy radicals [59]. It was also identified that TRES is stable in acidic media up to pH 6, as, in acidic media, the hydroxyl group of TRES is protected from radical oxidation by the positively charged H_3_O^+^ [47]. Thus, TRES dissociates in neutral and basic media because of basic hydrolysis [60], and, in alkaline media, the dissociation of TRES follows first-order kinetics [47]. Overall, the ionization of TRES begins with the increasing pH in basic media, which marks the instability of the TRES [60].

Similar to formulation F14, the release profile of the free TRES drug was also assessed in water (pH 7) and acetate buffer (pH 5.4). The delayed release of the drug was observed in NLC formulations; this was markedly visible at the 3 h time point, where free TRES peaked to ~76% of the total drug concentration, whereas the NLC formulation had released ~37% (pH 5.4). This reduction is beneficial when considering the rapid metabolism of TRES (half-life of 1–2 h), potentially reducing the dosing frequency significantly. The present study demonstrates that the release of TRES is pH-dependent. Similar findings were also reported in the literature [47], where the TRES drug dissociation rate was observed in alkaline conditions; however, other researchers indicated the stability of TRES in acidic media [61,62,63]. Hence, it was found that TRES, as an anti-cancer drug, has better stability and sustained release in acidic media (i.e., pH 5) compared to pH 7 (causing drug degradation) [47]. Furthermore, the stability of TRES in pH 5 is preferable to target pulmonary system, since tumor growth causes the pH of the lungs to become acidic due to the excessive production of lactic acid, making it a suitable environment for TRES stability and sustained release [64].

## 4. Conclusions

In this study, 24 TRES-NLC formulations for pulmonary drug delivery were successfully prepared and optimized using a novel combination of solid lipid (Dynasan 116), liquid lipids (Capryol 90, Lauroglycol 90, Miglyol 810, and Tributyrin), surfactant (Tween 80), and co-surfactant (SPC S-75) in various ratios using probe sonication. Upon initial analysis of the freshly prepared formulations and formulations stored at 25 °C for four weeks, TRES-NLC formulations F8, F14, and F22 were selected based on their smaller particle size, lower polydispersity, and higher drug entrapment. The solid lipid type was deemed to not affect the physiochemical properties of the formulation. Contrastingly, the liquid lipid type, surfactant, and their ratios were noted to significantly influence the aforementioned formulation properties. After nebulization using the air-jet and ultrasonic nebulizers in the NGI stages, the F14 particles were consistent and smaller compared to the F8 and F22 formulations. Additionally, the F14 formulation demonstrated higher stability at 4 and 25 °C for six months, indicating a more appropriate combination of excipients for potential drug delivery when compared to the F8 and F22 formulations. This suggests that Dynasan 116 as the solid lipid, Miglyol 810 as the liquid lipid, and Tween 80 as the surfactant in formulation F14 are a promising combination that prevents particle aggregation/fusion and provides long-term storage stability. Upon comparison of the air-jet nebulizer with the ultrasonic nebulizer by employing formulation F14, the air-jet nebulizer exhibited lower amounts of TRES drug deposition in the initial stages and higher in the middle to later stages of the NGI. The air-jet nebulizer, although exhibiting prolonged nebulization, delivered higher mass output (retained less formulation F14 in the nebulizer reservoir, attributed to the mechanism of aerosol generation and the design of the nebulizer). During nebulization, the air-jet nebulizer exhibited superior performance in terms of higher ED, FPD, FPF, and RF when compared to the ultrasonic nebulizer using formulation F14. Moreover, the F14 formulation and TRES as a free drug showed higher sustained release and stability in acetate buffer. Thus, the air-jet nebulizer is suggested to be a significantly superior nebulizer when using TRES-NLC formulation F14 for pulmonary drug delivery.

## Figures and Tables

**Table 1:** Composition of TRES-NLC formulations consisting of one solid lipid (Dynasan 116) and four liquid lipids (Capryol 90, Lauroglycol 90, Miglyol 810, and Tributyrin) in three different weight ratios, 10:90 (200 mg:1800 mg), 50:50 (1000 mg:1000 mg), or 90:10 (1800 mg:200 mg), where Tween 80 was used in two concentration (0.5% (250 mg) or 1.5% (750 mg)) as a surfactant and SPC-75 (50 mg) as a co-surfactant, and trans-resveratrol (TRES) (50 mg) was employed as the drug to prepare 24 TRES-NLC formulations.

Formulation	Solid Lipid (mg)	Liquid Lipid (mg)	Solid Lipid: Liquid Lipid (*w*/*w*)	Tween-80 (%)	Co-Surfactant (mg)	TRES (mg)
F1	Dynasan 116	Capryol 90	10:90	0.5	50	50
F2	Dynasan 116	Capryol 90	10:90	1.5	50	50
F3	Dynasan 116	Capryol 90	50:50	0.5	50	50
F4	Dynasan 116	Capryol 90	50:50	1.5	50	50
F5	Dynasan 116	Capryol 90	90:10	0.5	50	50
F6	Dynasan 116	Capryol 90	90:10	1.5	50	50
F7	Dynasan 116	Lauroglycol 90	10:90	0.5	50	50
F8	Dynasan 116	Lauroglycol 90	10:90	1.5	50	50
F9	Dynasan 116	Lauroglycol 90	50:50	0.5	50	50
F10	Dynasan 116	Lauroglycol 90	50:50	1.5	50	50
F11	Dynasan 116	Lauroglycol 90	90:10	0.5	50	50
F12	Dynasan 116	Lauroglycol 90	90:10	1.5	50	50
F13	Dynasan 116	Miglyol 810	10:90	0.5	50	50
F14	Dynasan 116	Miglyol 810	10:90	1.5	50	50
F15	Dynasan 116	Miglyol 810	50:50	0.5	50	50
F16	Dynasan 116	Miglyol 810	50:50	1.5	50	50
F17	Dynasan 116	Miglyol 810	90:10	0.5	50	50
F18	Dynasan 116	Miglyol 810	90:10	1.5	50	50
F19	Dynasan 116	Tributyrin	10:90	0.5	50	50
F20	Dynasan 116	Tributyrin	10:90	1.5	50	50
F21	Dynasan 116	Tributyrin	50:50	0.5	50	50
F22	Dynasan 116	Tributyrin	50:50	1.5	50	50
F23	Dynasan 116	Tributyrin	90:10	0.5	50	50
F24	Dynasan 116	Tributyrin	90:10	1.5	50	50

**Table 2:** Particle size, polydispersity index (PDI), zeta potential, and drug entrapment efficiency of the freshly prepared TRES-NLC formulations and those stored at 25 °C for four weeks. The molar ratios (drug to solid and liquid lipids) remained the same for freshly prepared TRES-NLC formulations and for those stored at 25 °C for four weeks. Data are reported as mean ± SD, n = 3.

Formulations	Size (nm)		PDI		Zeta Potential (mV)		Entrapment Efficiency (%)		Molar Ratios (Drug:Lipids)
	After Preparation	After Four Weeks	After Preparation	After Four Weeks	After Preparation	After Four Weeks	After Preparation	After Four Weeks	After Preparation and After Four Weeks
F1	245.46 ± 7.68	514.47 ± 8.75	0.25 ± 0.03	0.77 ± 0.18	−26.75 ± 4.77	−28.27 ± 5.12	97.15 ± 9.54	95.92 ± 7.82	0.02:1
F2	202.68 ± 8.52	258.72 ± 8.49	0.24 ± 0.08	0.31 ± 0.11	−26.35 ± 4.98	−30.85 ± 5.44	96.46 ± 7.25	95.17 ± 6.16	0.02:1
F3	272.13 ± 6.21	1059.70 ± 9.65	0.29 ± 0.11	0.38 ± 0.06	−25.60 ± 5.13	−28.15 ± 4.58	94.62 ± 6.58	93.08 ± 7.33	0.03:1
F4	224.67 ± 7.45	452.72 ± 8.12	0.25 ± 0.12	0.37 ± 0.05	−29.25 ± 6.95	−32.95 ± 5.08	94.62 ± 9.16	93.18 ± 6.74	0.03:1
F5	324.45 ± 7.44	817.75 ± 8.32	0.53 ± 0.09	0.43 ± 0.13	−26.45 ± 5.77	−27.35 ± 4.18	92.91 ± 5.49	91.07 ± 7.55	0.06:1
F6	310.15 ± 9.52	422.75 ± 7.54	0.46 ± 0.11	0.53 ± 0.04	−32.85 ± 5.34	−31.48 ± 6.58	91.16 ± 7.59	90.71 ± 6.49	0.06:1
F7	165.25 ± 8.25	202.25 ± 7.07	0.28 ± 0.03	0.20 ± 0.05	−32.18 ± 6.23	−31.03 ± 7.15	97.65 ± 8.25	98.22 ± 7.26	0.03:1
F8	136.48 ± 9.15	149.01 ± 9.73	0.19 ± 0.03	0.21 ± 0.05	−29.50 ± 4.42	−31.24 ± 6.72	97.73 ± 8.19	96.76 ± 6.59	0.03:1
F9	190.80 ± 8.55	231.25 ± 9.71	0.34 ± 0.08	0.25 ± 0.08	−36.15 ± 6.78	−34.67 ± 6.69	97.06 ± 6.23	96.16 ± 6.38	0.04:1
F10	163.80 ± 8.27	211.55 ± 7.92	0.29 ± 0.04	0.26 ± 0.05	−33.16 ± 6.62	−31.33 ± 5.92	95.19 ± 7.54	93.48 ± 7.09	0.04:1
F11	288.05 ± 6.79	516.25 ± 3.59	0.58 ± 0.27	0.41 ± 0.03	−25.52 ± 3.53	−25.80 ± 6.44	93.47 ± 6.85	91.28 ± 6.18	0.07:1
F12	206.05 ± 5.65	258.49 ± 7.29	0.44 ± 0.11	0.65 ± 0.33	−36.85 ± 6.69	−36.48 ± 7.06	93.82 ± 5.28	91.75 ± 6.68	0.07:1
F13	186.70 ± 4.78	254.23 ± 6.75	0.20 ± 0.05	0.25 ± 0.06	−30.15 ± 6.57	−32.39 ± 6.91	98.52 ± 5.97	97.55 ± 7.16	0.04:1
F14	142.41 ± 8.28	158.90 ± 7.99	0.15 ± 0.03	0.19 ± 0.11	−36.75 ± 6.86	−35.09 ± 7.98	98.17 ± 7.32	96.78 ± 5.91	0.04:1
F15	206.75 ± 7.25	298.76 ± 6.59	0.26 ± 0.06	0.34 ± 0.08	−30.60 ± 6.67	−29.07 ± 6.19	98.06 ± 4.16	95.72 ± 6.28	0.05:1
F16	182.80 ± 9.16	203.75 ± 7.47	0.21 ± 0.07	0.30 ± 0.09	−38.22 ± 7.36	−35.72 ± 6.69	96.24 ± 4.55	94.65 ± 5.53	0.05:1
F17	206.81 ± 7.26	492.25 ± 9.26	0.43 ± 0.02	0.24 ± 0.06	−34.81 ± 6.54	−31.65 ± 6.41	94.68 ± 5.62	92.26 ± 5.06	0.08:1
F18	187.19 ± 8.17	205.58 ± 9.48	0.28 ± 0.12	0.59 ± 0.25	−29.30 ± 7.97	−30.25 ± 6.98	94.55 ± 4.68	93.71 ± 6.26	0.07:1
F19	288.65 ± 7.63	346.77 ± 9.41	0.29 ± 0.06	0.32 ± 0.08	−25.85 ± 6.06	−29.45 ± 6.15	98.02 ± 5.05	98.82 ± 5.29	0.03:1
F20	265.70 ± 9.53	292.93 ± 8.09	0.25 ± 0.05	0.28 ± 0.04	−34.30 ± 4.68	−36.15 ± 6.46	98.48 ± 5.52	97.03 ± 5.18	0.03:1
F21	172.46 ± 6.55	379.95 ± 9.27	0.44 ± 0.09	0.32 ± 0.14	−30.25 ± 7.06	−32.65 ± 6.72	96.13 ± 4.87	98.27 ± 1.56	0.05:1
F22	144.54 ± 7.48	156.82 ± 8.39	0.17 ± 0.06	0.20 ± 0.03	−30.95 ± 6.88	−32.45 ± 7.70	97.34 ± 5.22	96.38 ± 4.51	0.05:1
F23	341.85 ± 7.84	393.65 ± 10.56	0.47 ± 0.05	0.44 ± 0.06	−30.42 ± 6.16	−27.15 ± 7.31	95.92 ± 5.72	94.27 ± 6.26	0.07:1
F24	202.59 ± 7.73	259.56 ± 8.62	0.39 ± 0.08	0.36 ± 0.11	−35.42 ± 6.67	−37.41 ± 5.86	95.44 ± 6.48	94.39 ± 4.71	0.07:1

**Table 3:** Particle size, polydispersity index (PDI), zeta potential, drug entrapment efficiency, and drug recovery of F8, F14, and F22 upon storage for 2, 4, or 6 months at 4 or 25 °C. Data are mean ± SD, n = 3.

Formulation	Storage Temperatures									
	4 °C					25 °C				
	Particle Size (nm)	PDI	Zeta Potential (mV)	Entrapment Efficiency (%)	Drug Recovery (%)	Particle Size (nm)	PDI	Zeta Potential (mV)	Entrapment Efficiency (%)	Drug Recovery (%)
2nd month										
F8	193.56 ± 7.74	0.32 ± 0.03	−27.83 ± 4.57	96.38 ± 5.75	94.52 ± 4.45	209.13 ± 6.65	0.38 ± 0.02	−23.72 ± 5.36	95.47 ± 4.12	93.40 ± 4.98
F14	175.26 ± 6.02	0.19 ± 0.03	−28.31 ± 6.44	97.22 ± 4.56	93.22 ± 4.97	206.46 ± 5.72	0.26 ± 0.03	−30.18 ± 6.16	96.12 ± 4.67	94.29 ± 3.10
F22	197.81 ± 7.28	0.24 ± 0.02	−30.05 ± 5.19	97.24 ± 4.01	94.41 ± 4.38	224.67 ± 7.69	0.32 ± 0.03	−26.84 ± 5.37	95.49 ± 4.09	96.27 ± 4.28
4th month										
F8	598.65 ± 9.16	0.36 ± 0.04	−25.92 ± 6.48	85.98 ± 5.36	78.37 ± 4.68	869.52 ± 9.26	0.38 ± 0.03	−29.82 ± 6.42	89.32 ± 3.59	67.97 ± 5.06
F14	196.35 ± 5.85	0.18 ± 0.03	−30.83 ± 5.71	94.51 ± 4.67	85.53 ± 5.46	224.68 ± 7.49	0.20 ± 0.04	−35.06 ± 6.55	95.79 ± 4.72	79.18 ± 4.88
F22	281.47 ± 8.09	0.42 ± 0.08	−35.28 ± 5.93	92.41 ± 4.18	68.97 ± 4.68	375.47 ± 7.67	0.46 ± 0.11	−34.87 ± 5.09	92.98 ± 4.21	55.19 ± 5.64
6th month										
F8	886.14 ± 7.55	0.41 ± 0.12	−25.38 ± 6.49	88.68 ± 4.15	60.04 ± 4.22	985.76 ± 7.16	0.42 ± 0.07	−28.52 ± 6.47	89.24 ± 4.55	55.78 ± 3.65
F14	199.64 ± 8.16	0.20 ± 0.06	−28.98 ± 5.71	95.38 ± 5.35	75.97 ± 4.31	245.45 ± 6.73	0.23 ± 0.06	−31.84 ± 5.46	94.89 ± 4.26	70.84 ± 4.52
F22	313.59 ± 7.34	0.51 ± 0.10	−31.46 ± 6.25	94.52 ± 4.43	52.16 ± 5.72	476.58 ± 6.92	0.46 ± 0.08	−30.44 ± 7.34	92.35 ± 5.17	45.43 ± 4.68

**Table 4:** Nebulization performance of air-jet and ultrasonic nebulizers employing TRES-NLC formulation F14 using NGI for emitted dose (ED), fine particle dose (FPD), fine particle fraction (FPF), and respirable fraction (RF). Data are mean ± SD, n = 3.

Characterization	Air-Jet Nebulizer	Ultrasonic Nebulizer
FPD (mg)	1.15 ± 0.05	0.78 ± 0.01
FPF (%)	24.74 ± 2.26	16.78 ± 1.38
RF (%)	94.05 ± 5.68	76.44 ± 3.75
ED (%)	80.95 ± 2.75	71.49 ± 2.06

## Data Availability

The original contributions presented in this study are included in the article. Further inquiries can be directed to the corresponding author.
